# How Can Hotel Employees Produce Workplace Environmentally Friendly Behavior? The Role of Leader, Corporate and Coworkers

**DOI:** 10.3389/fpsyg.2021.725170

**Published:** 2021-09-23

**Authors:** Shanting Zheng, Lin Jiang, Wenjing Cai, Binfeng Xu, Xiaopei Gao

**Affiliations:** ^1^ School of Tourism and Events, Hefei University, Hefei, China; ^2^ Intellectual Property Research Institute, University of Science and Technology of China, Hefei, China; ^3^ School of Public Affairs, University of Science and Technology of China, Hefei, China; ^4^ Department of Management & Organization, Vrije Universiteit Amsterdam, Amsterdam, Netherlands

**Keywords:** workplace environmentally friendly behavior, environmentally-specific servant leadership, perceived corporate environmental responsibility, coworkers’ work group green advocacy, green role identity

## Abstract

Although previous studies have acknowledged that leaders’ such environmental behaviors and environmental issues are becoming critical for long-term development, little research has focused on why, how and when perceived environmentally specific servant leadership contributes to employees’ workplace environmentally friendly behavior in the hotel industry. This paper aims to fill this research gap by using social identity theory to test employees’ green role identity as a mediator and their perceived corporate environmental responsibility and perceived coworkers’ work group green advocacy as moderators in the relationship between perceived environmentally-specific servant leadership and workplace environmentally friendly behavior. Using a sample of 527 leader-follower dyads from six hotels in mainland China at two points in time, we found that employees’ green role identity mediates the positive relationship between perceived environmentally specific servant leadership and employees’ workplace environmentally friendly behavior. Moreover, employees’ perceived corporate environmental responsibility and perceived coworkers’ work group green advocacy were found to positively moderate the relationship between perceived environmentally-specific servant leadership and green role identity and between green role identity and workplace environmentally friendly behavior, respectively. Theoretical and practical implications are discussed.

## Introduction

Environmental preservation has become a domain of critical importance in the service industry, especially because hotels that move toward sustainability in a green manner can improve maintenance and guest services (Chen and Chang, 2013). Compared to the environmental approaches in nonservice industries (e.g., public sectors), consumers with more awareness of their impact on the environment expect the hotels they interact with to do their part for the environment (Chung, 2020). Accordingly, considering that hotels face increasing pressure to pay more attention to environmental issues, “green hotels and green initiatives in hotels are quickly becoming the norm” (Arun et al., 2021, p. 2638). For example, most hotels have provided environmentally oriented guidelines to employees, which aim to develop and implement environmental initiatives throughout hotel companies (Chan et al., 2014). Given that employees play a key role in environmental hotels, scholars have investigated predictors that may contribute to developing employees’ green and environmental behaviors in the workplace. Specifically, a research stream suggests that since leaders and managers provide employees with direct supervision and guidance during their working hours (Vecchio and Boatwright, 2002; Ogunfowora et al., 2021), from the perspective of the leadership approach, employees’ environmentally oriented behaviors in the workplace are significantly influenced by their direct leaders. Accumulated evidence has consistently shown that leadership styles such as displaying and stressing environmental issues can encourage followers to generate environmental behaviors (Robertson and Barling, 2013; Cai et al., 2020; Wu et al., 2021). Notably, environmentally-specific servant leadership has recently attracted scholars’ attention in the service industry (Afsar et al., 2018; Tuan, 2019; Aboramadan et al., 2021) by indicating the specific characteristics of leaders who serve the community in an environmental manner. For example, Tuan (2020) recruited tour operators and showed that environmentally-specific servant leadership can effectively stimulate employees’ green creative outcomes.

However, limited research has been conducted to examine the potential influence of environmentally-specific servant leadership on employees’ specific environmental behaviors, such as workplace environmentally friendly behavior, particularly in hotel work settings. To fill this gap, this research examines the potential relationship between perceived environmentally-specific servant leadership and employees’ workplace environmentally friendly behavior by exploring the explanatory mechanisms (i.e., which mediators) and the boundary conditions (i.e., in the presence of which moderators).

Drawing on social identity theory (Ashforth and Mael, 1989), leaders can affect followers’ self-regulation of behavior by changing their self-identity (Lord et al., 1999; Chen et al., 2015). In relation to the green literature, when employees perceive an environmentally-specific servant leadership style that puts green value in the first place, they will pay more attention to green value, form their green role identity, and then provide high-quality service in an environmental manner. Therefore, we propose that employees’ green role identity plays a mediating role in the relationship between perceived environmentally-specific servant leadership and employees’ workplace environmentally friendly behavior.

**Figure 1:**
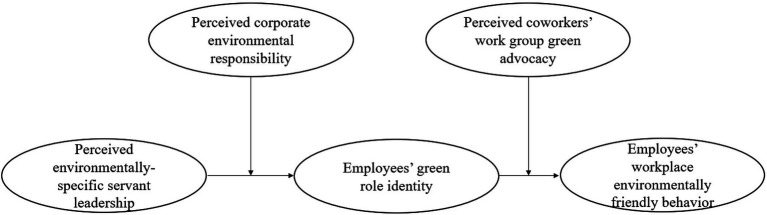
The hypothesized model.

Furthermore, considering that the working context (e.g., organizational green policy and coworkers’ green activities) may motivate individuals to behave in an expected way (Yoshida et al., 2014), we used social identity theory to identify the contingent role of two specific contextual factors, perceived corporate environmental responsibility and perceived coworkers’ work group green advocacy, on the indirect relationship between perceived environmentally-specific servant leadership and employees’ workplace environmentally friendly behavior *via* green role identity. Specifically, since corporate environmental responsibility exerts a strong impact on employees’ perception of their employers, when employees perceive that their hotels are enacting various activities of corporate environmental responsibility, they are more likely to receive signals about the value of being environmental in the workplace (Dögl and Holtbrügge, 2014; Ruepert et al., 2017). Consequently, their identity of being green increases. Moreover, research findings from previous studies have indicated that coworkers’ behavior can directly or indirectly impact employees’ relevant behaviors through personal interactions (Kidwell et al., 1997; Afsar and Umrani, 2020). For example, Kim et al. (2017) found that colleagues’ green advocacy shapes employees’ social interactions through open discussion of environmental sustainability, shared environmental knowledge, and the communication of their views to encourage employees to engage in eco-friendly behavior. When employees perceive that their coworkers are environmentally friendly at work and believe that their coworkers’ green advocacy is positive, they are more likely to engage in sustainable behaviors (Tian et al., 2020). Following this line of reasoning, when employees recognize their perception of coworkers’ work group green advocacy, they tend to strengthen their green identity to behave in a more environmentally friendly way. Figure 1 shows the hypothesized model in the current study.

By investigating the mediating role of employees’ green role identity and the moderating role of both perceived corporate environmental responsibility and perceived coworkers’ work group green advocacy in the association between perceived environmentally-specific servant leadership and employees’ workplace environmentally friendly behavior, the current study aims to contribute to the green literature in the following ways. First, this study fills the research linking perceived environmentally-specific servant leadership and employees’ workplace environmentally friendly behavior in the service industry, which enriches the current understanding of leadership approaches as predictors that facilitate followers’ desirable outcomes in terms of green and environmental behaviors in the workplace. Second, we open the black box of the association between perceived environmentally-specific servant leadership and employees’ workplace environmentally friendly behavior to advance scientific understanding of the identification lens of leaders’ desirable approach toward followers’ corresponding outputs. This also helps practitioners develop and use effective leadership interventions. Finally, by identifying two different contextual moderators in terms of employees’ perceptions of their organization and coworkers’ green-related characteristics, we contribute to the limited research that explicitly points to the fact that a green-related working environment can strengthen staff’s green endeavors. In this vein, our findings address a recent call for investigation that acknowledges the boundary condition of organizational and coworkers’ attitudes, norms and actions as they affect employees (Tariq et al., 2016).

## Theory and Hypotheses

### Perceived Environmentally-Specific Servant Leadership and Workplace Environmentally Friendly Behavior

In recent years, environmental issues have attracted great attention from researchers and organizations (Go et al., 2020; Yılmaz and Anasori, 2021). Effectively solving environmental issues has become a major external challenge facing organizations to improve their strategic position (Afsar et al., 2018). In response to environmental sustainability, researchers have identified environmentally friendly behavior as a factor that positively affects employees’ adherence to environmental concerns (Lee et al., 2013; Chiu et al., 2014; Han, 2015). Employees’ workplace environmentally friendly behavior emphasizes responsibility for the environment, which refers to the actions taken by employees to protect the environment and commit to solving environmental problems (Luu, 2019). Previous studies on environmentally friendly behaviors and their driving factors have examined factors such as positive and negative emotions, stakeholder groups in tourism, and destination social responsibility (Cheng et al., 2013; Su and Swanson, 2017). However, these studies mainly focus on the factors that drive tourists’ environmentally friendly behavior. In addition, to further explore the factors of the environmentally friendly behavior of the members of the organization, heated discussions have been launched in academic circles. Luu (2019) found that environmentally specific servant leadership has an impact on OCB in the environment. Environmentally-specific servant leadership demonstrates the exemplary environmental responsibility behaviors of leaders and creates an organizational atmosphere that focuses on environmental impact, thereby motivating employees’ environmentally friendly behavior and initiative (Rodgers, 2010; Biron and Bamberger, 2012).

Environmentally-specific servant leaders refer to leaders who have environmental values and commitment to green goals (Tuan, 2018) and serve and help employees contribute to the sustainability of the organization and the larger community. Perceived environment-specific servant leadership is described as a leadership style in which followers perceive that their leader puts environmental interests above economic interests (Afsar et al., 2018). This kind of leadership focuses on cultivating employees’ environmental values (Tuan, 2018). The influence of the behavioral characteristics of environment-specific servant leadership makes employees believe that their leadership is a role model worthy of emulation. This positive influence can stimulate employees’ environmental motivation and psychological atmosphere for environmental protection. Employees are likely to take environmentally friendly actions under environment-specific servant leadership (Lee et al., 2013; Luu, 2019). From the perspective of the servant leadership attribute framework of Van Dierendonck (2011), environment-specific servant leadership can make employees become environmental citizens by providing direction and authorization to employees. Perceived environment-specific servant leadership can be used as a source of green-related resources for the team and its members. By shaping the green climate of the entire team, team members are more inclined to invest their current green-related resources in environmentally friendly activities (Dumont et al., 2017; Ye et al., 2019).

### Employees’ Green Role Identity as a Mediator

Role identity refers to the self-component corresponding to the social role we play. The number of identities is limited only by the number of structural role relationships in which a person is involved (Stryker, 1980; Grube and Piliavin, 2000). When role identity meets the key requirement of self-verification, it motivates role performance (Markus and Wurf, 1987). The more important a person’s role identity is, the higher the probability that the person’s behavior is consistent with this identity (Stryker, 1980). Specifically, studies have shown that environmental self-identity is closely related to a series of environmental behavior indicators, including product selection and judgment of environmental dilemmas (Van der Werff et al., 2014). Another study defined the self-identity measure within the context of green consumers, which correlates with individuals’ sensitivity to being associated with “green issues” (Carfora et al., 2017), and a consumer with a higher self-concept of personality will be addicted to green buying behavior (Sharma et al., 2020). In addition, based on the theoretical argumentation of the role identity perspective (Burke, 1991) and research findings from Hobman and Frederiks (2014), green role identity can be viewed as an individual’s identification of him or herself as an environmentally-friendly person. An individual with a green role identity considers green activities as a salient component of their role and is inclined to be active and proactive in finding effective and creative solutions to environmental problems.

According to social identity theory (Ashforth and Mael, 1989), leaders’ behavior affects employees’ behavior by influencing employee identity (Herman and Chiu, 2014). Environmentally-specific servant leadership that puts environmental interests first is the source of green-related resources (such as green value) and instills these green resources into employees, prompting employees to internalize green value into their self-concepts and then develop employees’ green role identity (Duggleby et al., 2009). In addition, a study by Van der Werff et al. (2014) found that green values shape environmental self-identity. A large number of studies have shown that perceived environmentally-specific servant leadership can meet employees’ psychological needs, such as role identity recognition (Yoshida et al., 2014) and cultivating service-oriented role recognition (Zhao et al., 2016). Therefore, under the influence of perceived environmentally-specific servant leadership, employees will realize the importance of green behavior, pursue green value, and then develop a green role identity (Duggleby et al., 2009). Employees with high green role recognition tend to make better decisions on green solutions and are more inclined to strive to control related resources and take environmental protection actions to meet the expectations of the role (Koseoglu et al., 2017).

Based on the reasoning above, we expect that under the influence of perceived environmentally-specific servant leadership, employees can realize the necessity and value of green activities in the workplace and form a green role identity. Consequently, they are more likely to adopt workplace environmentally friendly behavior. In conclusion, we propose the following hypothesis:

Hypothesis 1: Employees’ green role identity mediates the relationship between perceived environmentally-specific servant leadership and followers’ workplace environmentally friendly behavior.

### Perceived Corporate Environmental Responsibility as a Moderator

Corporate environmental responsibility refers to the actions taken by companies to achieve environmental sustainability in compliance with environmental ethics and legal requirements (Lindgreen et al., 2009). For a company, the realization of a green strategy and culture is an important activity that shows its environmental responsibility (Dögl and Holtbrügge, 2014), which is manifested in actively encouraging employees to participate in environmental protection activities and can be evaluated and rewarded through environmental performance standards employees (Jackson et al., 2011). According to social identity theory, environmentally specific servant leadership puts the green value of the enterprise at the core of the development of the enterprise and strengthens efforts to cultivate green values for employees. In this type of enterprise, perceived environmentally-specific servant leadership encourages employees to internalize green values into their own self-concepts for better development and develop themselves as environmentally friendly people or green role identity (Luu, 2019). In this process, a company with a strong sense of environmental responsibility will be more likely to promote employees’ green organizational identity (Song et al., 2019). Specifically, when employees perceive that the hotel is implementing various corporate environmental responsibility activities, they are more likely to receive information about the value of the workplace environment. Therefore, their green identity may increase. Taken together, perceived corporate environmental responsibility is a facilitator that positively strengthens the relationship between perceived environmentally-specific servant leadership and followers’ green role identity. Thus, we formulate the following hypothesis:

Hypothesis 2: Perceived corporate environmental responsibility positively moderates the relationship between perceived environmentally-specific servant leadership and followers’ green role identity, such that the relationship is stronger when employees’ perceived corporate environmental responsibility is high rather than low.

### Perceived Coworkers’ Work Group Green Advocacy as a Moderator

Coworkers’ Work Group Green Advocacy means that colleagues actively discuss and share effective environmental knowledge, ecological problems and possible solutions to further improve environmentally friendly behavior (Cherian and Jacob, 2012; López-Mosquera et al., 2015). Previous studies have shown that work group members’ perceptions of the work environment can influence employees’ related behaviors (Kidwell et al., 1997) through social interaction (Klein et al., 2001). For example, under the influence of leaders’ green values, work group members will be more active in advocating green behaviors to strengthen the relationship between the work group and their leaders, and the more employees can recognize their green identity and do things in a more environmentally friendly way (Cialdini et al., 1990; Kim et al., 2017). Coworkers’ Work Group Green Advocacy requires colleagues to discuss environmental problems and possible solutions, share relevant knowledge, and try to improve the environment through communication, which can positively affect the environmental behavior of others (Norton et al., 2015; Shah et al., 2021). According to social identity theory, when employees more perceive their coworkers’ work group green advocacy, they will strengthen their green identity and adopt a more environmentally friendly working style. Taken together, perceived coworkers’ work group green advocacy is a facilitator that positively strengthens the relationship between employees’ green role identity and their workplace environmentally friendly behavior. Thus, we formulate the following hypothesis:

Hypothesis 3: Perceived coworkers’ work group green advocacy positively moderates the relationship between employees’ green role identity and their workplace environmentally friendly behavior, such that the relationship is stronger when coworkers’ work group green advocacy is high rather than low.

## Materials and Methods

### Sample and Procedure

We used the survey questionnaire to collect data in China. Specifically, because green and environmental issues are becoming critically important in the current service industry, especially in hotels, we invited employees and their direct supervisors to complete questionnaires regarding green and environmental practices. To avoid the problem of causality, we employed a time-lagged research design with a 1-month time interval. Before submitting questionnaires, we first randomly selected 10 hotels with an established green strategy in a middle city in mainland China. We then contacted the HR departments of these hotels by asking them whether they were willing to participate in our study. After receiving their conformation of participation from six hotels, one of the authors, with the help of the manager in the HR department, visited these hotels to ask employees who were willing to join in the survey study and then received 679 responses of conformation. In the following, the author submitted the questionnaires to these employees at Time 1. These employees provided their demographic information as well as information on their perception of their leaders’ environmentally-specific servant leadership, their green role identity, their perceived corporate environmental responsibility, and their perceived coworkers’ work group green advocacy. After deleting the invalid responses with missing information, we received 631 usable responses. One month later, at Time 2, we submitted the other questionnaire to these employees’ direct supervisors, who were asked to rate their followers’ workplace environmentally friendly behavior. The final sample contains 527 employees and 355 direct supervisors. Of the final sample of employees, 527 employees (64.2%) were female, their average age was 29.24years (SD=7.15), and their average organizational tenure was 5.38years (SD=4.07).

### Measures

#### Perceived Environmentally-Specific Servant Leadership

Following previous studies that operationalized perceived environmentally-specific servant leadership as an individual-level construct since it represents each follower’s perception about his or her direct leader’s behaviors and attitudes in terms of environmentally-specific servant leadership (Tuan, 2020), we used the 12-item scale from Tuan (2018) to measure perceived environmentally-specific servant leadership at Time 1 (e.g., “I am encouraged by my manager to volunteer in environmental activities.”; Cronbach *α*=0.93). The KMO value was 0.85, with the Bartlett test of sphericity achieving statistical significance (*p*<0.01).

#### Employees’ Green Role Identity

The three-item scale from Farmer et al. (2003) was used to assess green role identity at Time 1 (e.g., “To be a green employee is an important part of my identity.”; Cronbach *α*=0.77). The KMO value was 0.67, with the Bartlett test of sphericity achieving statistical significance (*p*<0.01).

#### Perceived Corporate Environmental Responsibility

We asked about perceived corporate environmental responsibility with three items from Ruepert et al. (2017) at Time 1 (e.g., “My organization has implemented policy and procedures to minimalize its impact on the environment.”; Cronbach *α*=0.97). The KMO value was 0.78, with the Bartlett test of sphericity achieving statistical significance (*p*<0.01).

#### Perceived Coworkers’ Work Group Green Advocacy

We assessed perceived coworkers’ work group green advocacy with three items from Kim et al. (2017) at Time 1 (e.g., “My coworkers share knowledge, information, and suggestions on workplace pollution prevention with other group members.”; Cronbach *α*=0.78). The KMO value was 0.70, with the Bartlett test of sphericity achieving statistical significance (*p*<0.01).

#### Employees’ Workplace Environmentally Friendly Behavior

At Time 2, employees’ workplace environmentally friendly behavior was measured with four items from Saifulina and Carballo-Penela (2017) (e.g., “This employee turns lights off when not in use.”; Cronbach *α*=0.97). The KMO value was 0.67, with the Bartlett test of sphericity achieving statistical significance (*p*<0.01).

#### Control Variables

Previous studies have indicated the influences of gender on employees’ pro-environmental behaviors (e.g., women tend to behave more environmentally; Vicente-Molina et al., 2018); therefore, we controlled for gender (1=male; 2=female) in the present study. In addition, considering that elderly individuals may be more concerned about the betterment of the environment through displaying green activities (Biswas et al., 2021), we controlled employees’ age (in years). Since a recent review shows that when employees have a higher level of education, their environmental behaviors would arise (Lu et al., 2017), we control participants’ educational level (1=High school/technical school and below; 2=Bachelor’s degree; and 3=Master’s degree and above). Finally, given the research evidence that tenure is related to individuals’ green behavior (Kim et al., 2017), participants’ working tenure (1=less than 1year; 2=from 1 to 5years; 3=from 6 to 10years; and 4=more than 10years) was controlled.

### Analytical Strategy

To test mediation and moderation effects in the current study (i.e., H1, H2, and H3), we used SPSS 25.0 to test hypotheses using separate hierarchical multiple regression analyses. To further clarify the mediation effect, we employed the PROCESS program developed by Hayes (Preacher et al., 2007) in SPSS using a bootstrap procedure with 5,000 samples to produce a confidence interval (CI) for the indirect effect. Next, to test the moderated mediation effect, we employed PROCESS using the Model 21 template to obtain bias-corrected bootstrapped confidence intervals for the conditional indirect effect. Specifically, we also bootstrapped with 5,000 iterations to generate bias-corrected CIs for the significance tests of the conditional indirect effects (95% CIs) in the moderated mediation models (Hayes, 2013).

## Results

### Confirmatory Factor Analysis and Validity

To validate the developed constructs, a measurement model was estimated with a confirmatory factor analysis (CFA) in which each measurement item was loaded on its proposed constructs, and the constructs were allowed to be correlated in the analysis (Anderson and Gerbing, 1988). Using AMOS 22.0, we present the CFA results in Table 1. Specifically, the hypothesized model indices indicated acceptable fit: *χ*^2^=245.29, *df*=113, RMSEA=0.06, CFI=0.97, TLI=0.97. Furthermore, we compared our measurement model to four alternatives: (1) a four-factor model with perceived corporate environmental responsibility and perceived coworkers’ work group green advocacy combined, which fit worse than the hypothesized model, with *χ*^2^=757.03, *df*=119, RMSEA=1.00, CFI=0.85, TLI=0.85; (2) a three-factor model with perceived environmentally-specific servant leadership, perceived corporate environmental responsibility, and perceived coworkers’ work group green advocacy combined, which provided a worse fit than the hypothesized model, with *χ*^2^=1651.32, *df*=123, RMSEA=1.27, CFI=0.79, TLI=0.78; (3) a two-factor model with perceived environmentally-specific servant leadership, employees’ green role identity, perceived corporate environmental responsibility, and perceived coworkers’ work group green advocacy combined, providing a worse fit than our measurement model, with *χ*^2^=1789.20, *df*=127, RMSEA=1.35, CFI=0.66, TLI=0.66; and (4) a one-factor model with all factors combined, providing a worse fit than our measurement model with the combined model, with *χ*^2^=1834.11, *df*=130, RMSEA=1.38, CFI=0.59, TLI=0.59. These results indicated that the five constructs captured distinctiveness, as expected in the present study.

**Table 1:** Results of confirmatory factor analysis.

Models	*χ*^2^	Δ*χ*^2^	*df*	RMSEA	CFI	TLI
Hypothesized five-factor model	245.29	–	113	0.06	0.97	0.97
Four-factor model (perceived corporate environmental responsibility and perceived coworkers’ work group green advocacy combined)	757.03	511.74	119	1.00	0.85	0.85
Three-factor model (perceived environmentally-specific servant leadership, perceived corporate environmental responsibility, and perceived coworkers’ work group green advocacy combined)	1651.32	894.29	123	1.27	0.79	0.78
Two-factor model (perceived environmentally-specific servant leadership, employees’ green role identity, perceived corporate environmental responsibility, and perceived coworkers’ work group green advocacy combined)	1789.20	137.88	127	1.35	0.66	0.66
One-factor model (all combined)	1834.11	44.91	130	1.38	0.59	0.59

Since the independent variable, the mediator, and the moderators were all measured by employees (i.e., one source), we employed explanatory factor analysis (Harman, 1976) to identify the potential for common method bias (CMB). The results showed that one factor accounted for 30.15%, which is below the accepted threshold of 40%. Thus, CMB is not a serious problem in our study.

### Descriptive Statistics and Intercorrelations

Table 2 shows the means, standard deviation, and correlations of all the measures. The results show that the relationship between perceived environmentally-specific servant leadership and employee workplace environmentally friendly behavior was significant (*β*=0.25, *p*<0.01). As discussed, perceived environmentally-specific servant leadership had a significant positive correlation with employees’ green role identity (*β*=0.51, *p*<0.01), and employees’ green role identity had a significant positive correlation with employee’s workplace environmentally friendly behavior (*β*=0.40, *p*<0.01). Perceived corporate environmental responsibility had a significant positive correlation with employees’ green role identity (*β*=0.90, *p*<0.01). Finally, perceived coworkers’ work group green advocacy also had a positive correlation with employee’s workplace environmentally friendly behavior (*β*=0.05, *p*>0.05), but the relationship was not significant.

**Table 2:** Descriptive statistics and correlations among variables.

Variables	Mean	SD	1	2	3	4	5
1. Perceived environmentally-specific servant leadership	4.57	1.02	(0.93)				
2. Employees’ workplace environmentally friendly behavior	5.17	0.94	0.25^**^	(0.85)			
3. Employees’ green role identity	4.63	1.09	0.509^**^	0.40^**^	(0.77)		
4. Perceived corporate environmental responsibility	5.078	1.18	0.429^**^	0.36^**^	0.90^**^	(0.97)	
5. Perceived coworkers’ work group green advocacy	4.11	1.27	0.42^**^	0.05	0.32^**^	0.24^**^	(0.78)

*N*=527. Reliability coefficients are reported in parentheses on the diagonal.

* *p*<0.05;

** *p*<0.01.

### Hypothesis Tests

To test the hypothesis of whether employees’ green role identity mediates the impact of perceived environmentally-specific servant leadership on employees’ workplace environmentally friendly behavior, we used Model 4 in SPSS PROCESS (Hayes, 2013). The results indicate that the total effect of perceived environmentally-specific servant leadership on employees’ workplace environmentally friendly behavior was found to be significant (*β*=0.23, *t*=5.94, *p*<0.01). Moreover, the results in Table 3 show that the indirect effect was significant, with an indirect effect (*β*=0.17, SE=0.03) and a 95% confidence interval between 0.11 and 0.24, supporting H1.

**Table 3:** Direct and indirect effects of grit on creativity.

Direct effect			
Effect	SE	t	95% CI
0.06	0.04	1.43	[−0.02; 0.15]
Indirect effect			
Effect	Boot SE	Boot 95% CI	
0.17	0.03	[0.11; 0.24]	

*N*=527.

**Figure 2:**
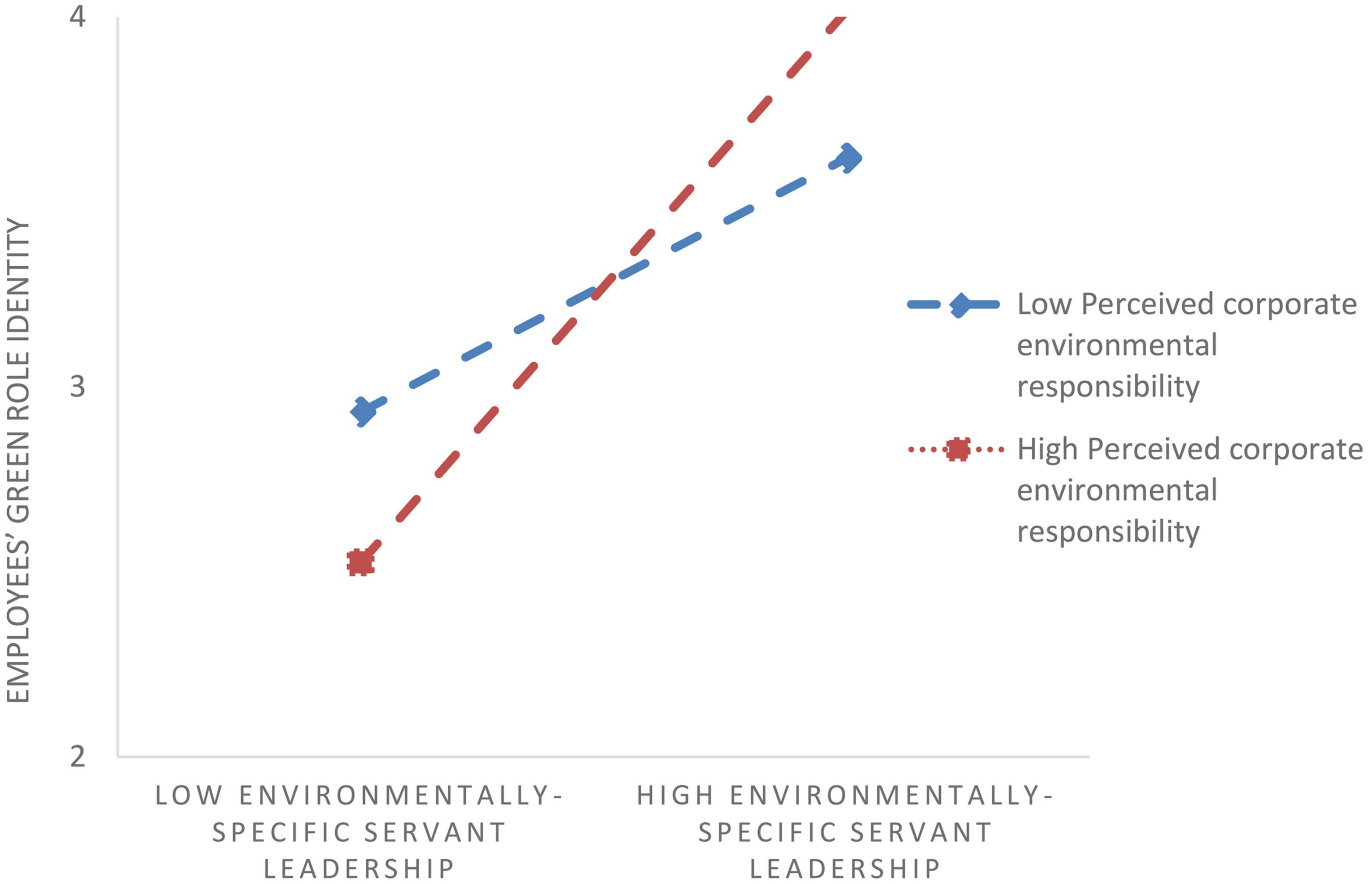
Interaction between environmentally-specific servant leadership and perceived corporate environmental responsibility in predicting employees’ green role identity.

To test H2, we introduce an interaction term (i.e., perceived environmentally-specific servant leadership×perceived corporate environmental responsibility) into our regression model. To test stage one moderated mediation, we used Model 7 in SPSS PROCESS (Hayes, 2013). Table 4 presents the results. Specifically, the interaction term is positively related to employees’ green role identity (*β*=0.08, *p*<0.01). We also illustrate the pattern of the interaction effect in Figure 2 to display the plot of the moderation effect. It shows that perceived corporate environmental responsibility significantly strengthens the relation between perceived environmentally-specific servant leadership and employees’ green role identity. We further conduct a simple slope test. Specifically, simple slope analyses showed that perceived environmentally-specific servant leadership was significantly related to employees’ green role identity at both high levels (simple slope=0.23, SE=0.03, *t*=8.92, *p*<0.01) and low levels (simple slope=0.09, SE=0.03, *t*=3.45, *p*<0.01) of perceived corporate environmental responsibility. H2 is thus supported.

**Table 4:** Results of moderated multiple regression analysis for employees’ green role identity.

Variable(s) entered	Model 1	Model 2	Model 3
Gender	−0.15^**^	−0.03	−0.03
Age	−0.03	−0.02	−0.01
Education	−0.06	−0.05^*^	−0.045^*^
Organizational tenure	0.11^*^	−0.00	−0.00
Perceived environmentally-specific servant leadership		0.150^**^	0.15^**^
Perceived corporate environmental responsibility		0.829^**^	0.83^**^
Environmentally-specific servant leadership×perceived corporate environmental responsibility			0.08^**^
Δ*R*^2^	0.04	0.79	0.01

*N*=527. Final model statistics: *F*=383.01, *R*^2^=0.84.

* *p*<0.05;

** *p*<0.01.

**Figure 3:**
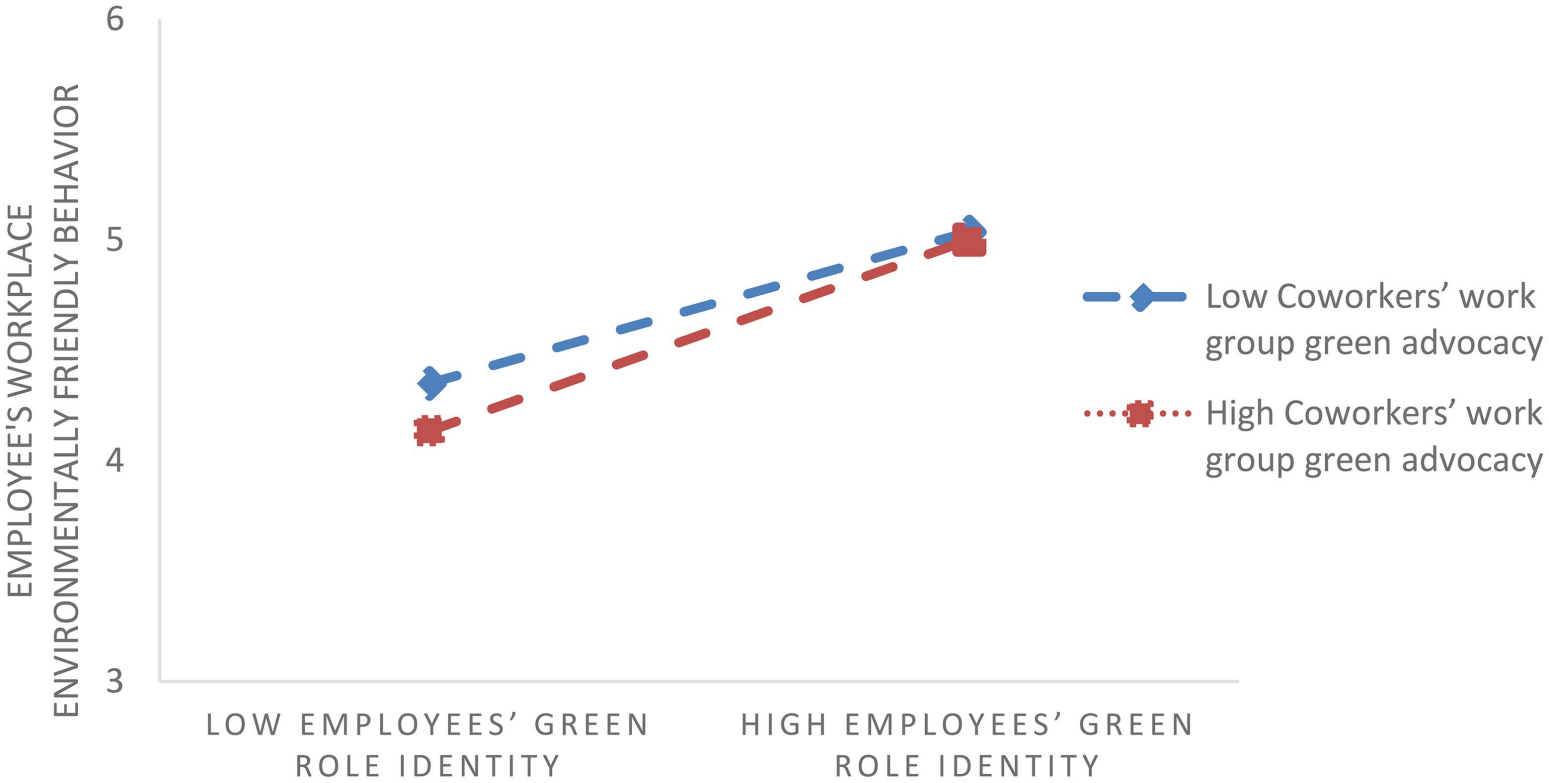
Interaction between employees’ green role identity and coworkers’ work group green advocacy in predicting employees’ workplace environmentally friendly behavior.

H3 predicted that perceived coworkers’ work group green advocacy moderates the relationship between employees’ green role identity and employees’ workplace environmentally friendly behavior. To test stage two moderated mediation, as mentioned in our theoretical diagram, we used Model 14 in SPSS PROCESS (Hayes, 2013). Specifically, we estimated the conditional indirect effect of perceived environmentally-specific servant leadership on employee’s workplace environmentally friendly behavior through employees’ green role identity with different levels of coworkers’ work group green advocacy using unstandardized coefficients and bootstrapping with 5,000 samples to place 95% confidence intervals around estimates of the indirect effects. As shown in Figure 3, the indirect effect of perceived environmentally-specific servant leadership on employees’ workplace environmentally friendly behavior through employees’ green role identity was significantly increased both when perceived coworkers’ work group green advocacy was at a high level [indirect effect =0.46, 95% CI (0.36; 0.55)] and when perceived coworkers’ work group green advocacy was at a low level [indirect effect=0.228, 95% CI (0.13; 0.32)], as indicated by the significant interaction between employees’ green role identity and perceived coworkers’ work group green advocacy (*β*=0.18, *p*<0.01). Thus, H3 was supported.

Finally, to test the full model with employees’ green role identity mediating the effects of perceived environmentally-specific servant leadership on employees’ workplace environmentally friendly behavior, perceived corporate environmental responsibility moderating the effects of perceived environmentally-specific servant leadership on employees’ green role identity, and perceived coworkers’ work group green advocacy moderating the effects of employees’ green role identity on employees’ workplace environmentally friendly behavior, we used Model 21 in SPSS PROCESS (Hayes, 2013). Specifically, we estimated the conditional indirect effect of perceived environmentally-specific servant leadership on employee’s workplace environmentally friendly behavior through employees’ green role identity both in high perceived corporate environmental responsibility and low perceived corporate environmental responsibility and high coworkers’ work group green advocacy and low coworkers’ work group green advocacy using unstandardized coefficients and bootstrapping with 5,000 resamples to place 95% confidence intervals around estimates of the indirect effects. As shown in Tables 4–6, we found significant interactions between environmentally-specific servant leadership and perceived corporate environmental responsibility in predicting employees’ green role identity (*β*=0.08, *p*<0.01) and between employees’ green role identity and coworkers’ work group green advocacy in predicting employees’ workplace environmentally friendly behavior (*β*=0.18, *p*<0.01), providing evidence of moderated mediation at two different points along the causal chain. Thus, the results supported our hypothesized model.

**Table 5:** Results of moderated multiple regression analysis for employees’ workplace environmentally friendly behavior.

Step	Variable(s) entered	Model1	Model2	Model3	Model4
1	Gender	−0.01	0.04	0.05	0.05
	Age	−0.01	0.00	−0.00	−0.01
	Education	−0.03	−0.02	−0.01	0.00
	Organizational tenure	0.01	−0.03	−0.04	−0.03
2	Perceived environmentally-specific servant leadership		0.12^**^	0.11^*^	0.09
	Perceived corporate environmental responsibility		0.32^**^	0.00	0.06
3	Employees’ green role identity			0.39^**^	0.35^**^
	Perceived coworkers’ work group green advocacy			−0.12^**^	−0.17^**^
4	Employees’ green role identity×perceived coworkers’ work group green advocacy				0.18^**^
	*R*^2^	0.00	0.15	0.18	0.20

*N*=527. Final model statistics: *F*=16.53.

* *p*<0.05;

** *p*<0.01.

**Table 6:** Bootstrap results for the conditional indirect effects.

Condition	Indirect effect	Boot SE	95% CI
Low perceived corporate environmental responsibility, low perceived coworkers’ work group green advocacy	0.02	0.01	[0.003; 0.045]
High perceived corporate environmental responsibility, low perceived coworkers’ work group green advocacy	0.05	0.02	[0.02; 0.09]
Low perceived corporate environmental responsibility, high perceived coworkers’ work group green advocacy	0.04	0.02	[0.01; 0.07]
High perceived corporate environmental responsibility, high perceived coworkers’ work group green advocacy	0.10	0.02	[0.07; 0.15]

*N*=527.

## Discussion

### Theoretical Implications

The current study has several theoretical implications. First, we develop and examine a model of the potential association between perceived environmentally-specific servant leadership and employees’ workplace environmentally friendly behavior. That is, although previous research has indicated the potential benefits of an environmentally oriented leadership style in the workplace, limited empirical studies have been conducted to demonstrate why the specific leadership approach – i.e., followers’ perceived environmentally-specific servant leadership – can contribute to promoting employees’ workplace environmentally friendly behavior. The results advance research on employees’ environmental outcomes associated with leaders who enact environmentally-specific servant leadership (Afsar et al., 2018; Aboramadan et al., 2021). In this vein, we support previous findings showing that perceived environmentally-specific servant leadership can contribute to employees’ desirable outcomes, especially corresponding environmental behavioral outcomes (Tuan, 2019, 2020). At the same time, this study extends the knowledge of leadership approaches as predictors that facilitate followers’ desirable outcomes in terms of green and environmental behaviors in the workplace (Aboramadan et al., 2021). Moreover, we enrich the current understanding of the benefits of perceived environmentally-specific servant leadership in hotel work settings. Thus, consistent with previous studies, the role of leaders who display an environmentally-specific servant leadership approach is emphasized in the environmental research domain.

Second, the findings of the present research indicate the mediating role of followers’ green role identity in transferring the effect of perceived environmentally-specific servant leadership. In doing so, we fill a research gap by considering an identity perspective. Existing research has capitalized on the motivational cognitive perspective to clarify that employees’ green behavior can be significantly developed beyond their roles, which overlooks the potential development of employees’ self-identity (Whitmarsh and O’Neill, 2010). The current study empirically justifies the green-related identity of employees to suggest that employees’ green role identity serves as a bridge linking perceived environmentally-specific servant leadership to employees’ workplace environmentally friendly behavior. Specifically, the mediating process of followers’ green role identity helps to explain the association between perceived environmentally-specific servant leadership and employees’ workplace environmentally friendly behavior, which advances scientific understanding of the influence of environmentally oriented servant leadership on followers’ related green behaviors in the workplace and helps practitioners develop and use effective leadership interventions.

Moreover, we extend the use of social identity theory in the environmental literature by theoretically introducing the identification lens (Dono et al., 2010; Fielding and Hornsey, 2016). That is, a desirable leadership style can help followers build corresponding identifications, which increases the likelihood of their engaging in supportive green behaviors in the workplace. Consistent with previous studies in which individual values aligned with the organization are expected to result in optimal employee outcomes (e.g., organizational identification), we move beyond these findings by specifically exploring the import mediator of self-identification in terms of green and environmental concerns – i.e., green role identity. Consequently, the results of our study contribute to the literature by treating employees’ green role identity as an important variable that connects environmentally oriented leadership and social identity theory in the hospitality context.

Finally, the present results also indicate the boundary conditions under which perceived environmentally-specific servant leadership leads to employees’ workplace environmentally friendly behavior *via* green role identity. Specifically, instead of exploring one moderator in an indirect relation, we empirically demonstrate two key contextual factors: employees’ perceptions of their organization and coworkers’ green-related characteristics. Although previous studies have conceptually and empirically acknowledged the contingent role of contextual factors, limited research has been conducted to explore multiple contexts in strengthening or weakening the leadership-employee green outcomes relationship. Our study identifies two different contextual moderators in terms of employees’ perceptions of their organization and coworkers’ green-related characteristics, which contribute to explicitly pointing to the importance of a green-related working environment. That is, we find that groups and colleagues, independent of managers, provide more opportunities for employees to follow their leaders’ environmental servant characteristics and their own identification with green issues to behave more environmentally. Thus, we contribute to the knowledge that environmentally-specific servant leadership may differ in employees’ perceptions of their organization and coworkers’ green-related characteristics (Dumont et al., 2017; Wu et al., 2021). In this way, we address scholars’ call to explore the boundary condition of organizational and coworkers’ attitudes, norms and actions as they affect employees (Tariq et al., 2016).

### Practical Implications

According to the findings in the present research, we provide some practical implications. First, managers are encouraged to display an environmentally-specific servant leadership approach during working time through role modeling and motivating followers’ identification with green issues. For example, organizations can not only promote managers who have the intention of displaying an environmentally-specific servant leadership style but also select leaders who provide environmental services to employees through personality tests. In addition, environmental-related activities such as green exploitation and green exploration learning can be stressed throughout organizations to help build employees’ green mindset. For example, rewards can be provided to employees who engage in green endeavors (e.g., saving water and recycling papers). In doing so, employees build an environmental habit of serving their customers.

Second, organizations (e.g., hotels) should attach importance to adopting a “green” philosophy in the workplace to build a desirable and sustainable environment. For example, organizations could set rules of being environmental in the workplace. Meanwhile, more resources should be invested in strengthening the enacting of corporate environmental responsibility to increase engagement in workplace environmentally friendly behavior. Finally, the role of coworkers’ green and environmental attitudes and behaviors is highly emphasized. For example, team leaders can offer some training courses on enacting environmental behaviors to team members and encourage interactive learning among coworkers.

### Limitations

There are some limitations in the current study. First, although we conducted a time-lagged research design to collect data, future studies are highly encouraged to employ other research designs (e.g., a longitudinal research design) to determine the direction of causality among the variables. Furthermore, although our research focused on the workplace in hotels, we collected data only in the Chinese context of hotels; thus, it is highly recommended that other studies be conducted with different samples (e.g., employees working in the airline industry) to generalize the results reported in the present study.

Third, as noted in the second point, the sample of Chinese employees in the current research may reflect the deep-seated importance of dyadic relations in Chinese culture (Chen et al., 2002), which is inferentially traceable to Confucianism (Farh and Cheng, 2000). Thus, cultural-specific factors related to relational roles and accompanying obligations may have promoted followers’ identification with the leader and engagement in related green and environmental behaviors in response to environmental servant leadership among the participants in our research sample. Therefore, future research could consider some cultural characteristics to support the validity of the findings.

Finally, although we conceptualized and operationalized perceived environmentally-specific servant leadership as an individual-level construct in the current study because we followed existing research on each follower’s perception about his or her direct supervisor’s environmentally-specific servant leadership approach, some studies have suggested this as a team- or group-level predictor by using environmentally-specific servant leadership. Therefore, future research is encouraged to replicate our findings by using multilevel path analysis to examine the proposed model simultaneously.

## Conclusion

This study empirically examined the relationship between perceived environmentally-specific servant leadership and followers’ workplace environmentally friendly behavior and proposed the mediating effect of employees’ green role identity. Moreover, the moderating role of perceived corporate environmental responsibility and perceived coworkers’ work group green advocacy on this indirect relationship was confirmed in this study. The research findings shed light on the theoretical implications of the association between perceived environmentally-specific servant leadership and followers’ workplace environmentally friendly behavior. Service organizations such as hotels should begin to focus on environmentally friendly practices by encouraging managers to enact environmentally-specific servant leadership styles and engaging corporate social responsibility initiatives.

## Data Availability Statement

The raw data supporting the conclusions of this article will be made available by the authors, without undue reservation.

## Author Contributions

SZ and WC: conceptualization. LJ: methodology and visualization. LJ and WC: software and formal analysis. SZ, BX, and XG: validation. SZ: resources. SZ, WC, LJ, BX, and XG: writing – original draft preparation and writing – review and editing. BX and XG: project administration. SZ, WC, BX, and XG: funding acquisition. All authors contributed to the article and approved the submitted version.

## Funding

This research was funded by USTC Funding for Featured Liberal Arts, grant number YD2160002004; the Funds of S&T Innovation Strategy and Soft Science Research in Hefei, grant number 2020014; the Funds of S&T Innovation Strategy and Soft Science Research in Anhui Province, grant number 202006f01050007; the USTC Research Funds of the Double First-Class Initiative, grant number YD2160002010; the Fundamental Research Funds for The Central Universities, grant number WK2160000014; and the 2020 Provincial Quality Engineering Project of Online and Off-line Mixed Course, grant number 2020xsxxkc396.

## Conflict of Interest

The authors declare that the research was conducted in the absence of any commercial or financial relationships that could be construed as a potential conflict of interest.

## Publisher’s Note

All claims expressed in this article are solely those of the authors and do not necessarily represent those of their affiliated organizations, or those of the publisher, the editors and the reviewers. Any product that may be evaluated in this article, or claim that may be made by its manufacturer, is not guaranteed or endorsed by the publisher.
